# A Review of Alternative Controls for House Flies

**DOI:** 10.3390/insects12111042

**Published:** 2021-11-20

**Authors:** Nancy C. Hinkle, Jerome A. Hogsette

**Affiliations:** 1 Department of Entomology, University of Georgia, Athens, GA 30602, USA; 2 USDA-ARS-CMAVE, Gainesville, FL 32608, USA; Jerry.Hogsette@usda.gov

**Keywords:** *Musca domestica*, maggot, insect light trap, bait, air curtain, window screen, window trap, fly swatter, exclusion, biological control

## Abstract

**Simple Summary:**

People hate house flies, considering them both a nuisance and a health risk. Most fly suppression is based on insecticides, but more creative, environmentally friendly options exist and should be used when applicable. Cleaning up materials that attract flies, especially those producing enticing aromas, is a basic fly control strategy. Similarly, eliminating materials, e.g., animal wastes and garbage, in which maggots can develop is a critical means for reducing future adult populations. Preventing flies from entering buildings is the first line of defense, followed by efficacious methods for catching and killing flies that do manage to gain entry. Flies can be excluded from buildings with properly maintained air curtains, fans, slat doors, mesh screens on windows and doors, and by keeping entry doors closed. Potential fly harborage, e.g., vending machines, racks for newspapers or circulars, ash trays, shrubbery, or other fly resting sites, should not be situated near entry doors. If flies enter, they can be managed with ultraviolet light traps, window traps, window stickers, sticky tubes, sticky ribbons, insecticide sprays, baits, timed-release aerosols, and the classic fly swatter. In urban areas, fly management usually amounts to killing adult flies produced elsewhere faster than they arrive on site.

**Abstract:**

House flies are the most prevalent synanthropic pest worldwide. Although they seldom reproduce in homes, they invade buildings, cause annoyance, and carry pathogens. Urban pest management personnel are limited in their ability to locate and manage larval habitats, so most house fly management in urban settings focuses on adult fly suppression. Sanitation is probably the most critical component, eliminating odors that attract flies. Source reduction applies where larval habitats can be identified and eliminated. Exclusion involves keeping flies out of structures. Despite all efforts, flies will manage to enter the human environment, so exclusion includes air curtains, fans, screened windows, and doors. Ultraviolet light traps attract and immobilize, while window traps entice flies into devices that entrap them. Sticky tubes and ribbons rely on flies’ inclination to land on vertical lines to entangle them in glue. Even low-tech fly swatters can play significant roles in eliminating individual flies. Timed-release aerosol pyrethrin dispensers can be effective against flies confined in enclosed spaces. Toxic baits have limited use in urban settings. Chemical suppression remains a critical component of fly IPM, essential in situations requiring immediate fly elimination.

## 1. Introduction

House flies (*Musca domestica* L.) are pervasive pests, accompanying humans worldwide and serving as nuisances and vectors of pathogens. Their biology and behavior equip them for success, yielding large persistent populations that subvert every control method directed against them. Preferred house fly management involves suppressing immature stages before they develop wings and disperse. Larval habitats consist of moist organic material, constituting a limited space to target, while adult house flies can move long distances. Typically, house fly larval habitats are associated with agriculture, so urban pest management has limited opportunity to address source reduction. Of course, even in cities there are habitats suitable for house fly larval development such as garbage, wildlife carcasses, and feces, but these sites are not usually on properties subject to manipulation by the customer, leaving little recourse for larval suppression [1]. Pest management professionals (PMPs) therefore must focus their efforts on adult house flies.

The house fly is considered a filth fly because its immature stages develop in feces, carcasses, rotting garbage, and similar sites. House fly larvae use a range of habitats, so long as they are moist and contain suitable organic nutrients. Similarly, adult house flies use various foods and, due to their short developmental cycle and high reproductive rate, can rapidly increase their population numbers [2].

As a holometabolous insect, the house fly goes through egg, larval, pupal, and adult stages, lengths of which are temperature-dependent. The egg typically hatches in less than a day, three larval instars can be completed in about a week, and the adult emerges from the puparium about 5 days later. Under good conditions, egg to egg development can be 7 days, and this rapid generation turnover illustrates why house fly populations sometimes appear to explode [1]. A female house fly can produce 1000 eggs in her lifetime [3], in clutches of 100–150 eggs. Biotic and abiotic environmental factors prevent flies from reaching their reproductive potential.

**Figure 1:**
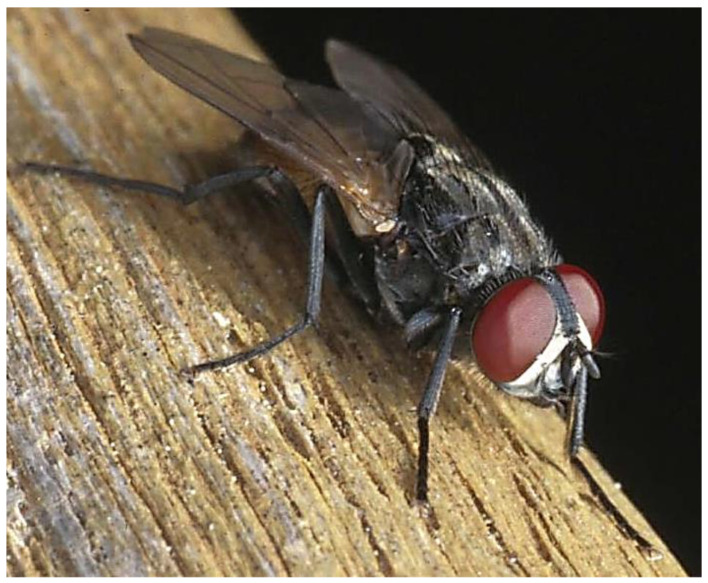
House flies are morphologically, physiologically, and behaviorally suited to serve as disease vectors.

House flies carry hundreds of species of pathogenic organisms including bacteria, fungi, and viruses [4]. Some transmission is mechanical, with disease organisms adhering to setae and other external structures, but some pathogens are consumed and multiply in the fly (Figure 1). Because flies not only walk on food, but defecate and regurgitate on it as well, there is ample opportunity for house flies to contaminate foodstuffs [5].

Flies are, of course, the original recyclers, decomposing many materials that humans find objectionable. Without flies and other coprophagous and necrophagous arthropods, humans would be wading through a fetid world. However, when winged adults emerge, flies become a threat to human health and comfort, and must be suppressed. Because of their prominence as a nuisance pest and because they can transmit human and animal disease agents, in some environments (such as a surgical suite) a single fly exceeds the action threshold.

### Need to Control Flies

Although health department regulations may provide incentive to manage house flies, the stronger impetus comes from dissatisfied customers. A single post to social media can affect business, so fly control is essential for brand reputation protection. Negative publicity can adversely impact a brand long after the problem has been resolved—or even if the company is found blameless; therefore, companies are highly motivated to prevent fly problems.

Urban house fly control is valued at over USD 1.87 billion annually in commercial accounts, with restaurants and facilities inspected by the Food and Drug Administration (FDA) (pharmaceutical/biotech and plants that process human and animal food) accounting for over half of the insect light trap (ILT) market. Most pest control companies lease the traps and charge for monthly service, which involves replacing glue boards, checking operation, cleaning the device, and replacing the bulb semi-annually. Considering the top ten markets use well over a million light traps, this involves substantial expenditure. Although residential accounts also compose a portion of the fly control market, there is no way to accurately estimate its annual economic value.

Although costs of house fly damage and control in U.S. agriculture are estimated at USD 450 million annually [2], that sum can be vastly increased by nuisance lawsuits, as was demonstrated in the USD 50 million settlement awarded against Smithfield Foods [6]. 

The internet is full of recommendations on killing or repelling flies, most of which have no factual basis and are certainly not supported by research results. Many of these are based on using common products found around the home such as herbal materials, the ever-popular apple cider vinegar, soaps, etc. The contention is that flies do not like strong odors such as essential oils and will be repelled by them, but there is no indication that any odor will keep a hungry fly away from its preferred food. Most of the natural compounds promoted for pest control have not been scientifically evaluated for either effectiveness or safety [7].

## 2. Integrated Pest Management (IPM) for Flies

Why are alternative fly controls of such interest? International trade highlights concern regarding overseas shipment and recipient refusal of insect contamination; thus, manufacturing and processing facilities are required to be fly-free. U.S. FDA regulations restrict fly presence in food and biotech product processing plants, so ILTs are used routinely in such premises. Fly resistance to traditional insecticides necessitates new alternatives, and house flies are notorious for the speed with which populations develop insecticide resistance to products with new modes of action [2]. The U.S. Environmental Protection Agency has registered few materials for house fly control in ‘sensitive sites’ such as around food and people. There is popular demand, because the public wants alternatives to what they perceive as ‘hard chemicals’. For a variety of reasons, it is desirable to expand available options for fly IPM.

Knowledge of the pest, its biology and behavior, are critical for determining the most effective means of suppression. For instance, nematodes are not effective in highly alkaline habitats [8]. As with all suppression efforts, some components are not compatible. For example, often parasitoids are more insecticide susceptible than is the host, so insecticide application precludes biological control with parasitoids.

Adult house fly control in sensitive environments (like food-handling facilities and hospitals) is challenging because both flies and insecticides are unwelcome, so alternative controls are needed. Fly management strategies include exclusion, indoor management, sanitation, and source reduction.

## 3. Exclusion

Exclusion from structures is the first line of defense in the management of house flies. Exclusion from buildings is of particular importance, especially if the buildings are food or medical facilities, but exclusion from structures that contain garbage or contaminated wastes from hospitals is also important for public health. In this section, exclusion techniques and their specific uses will be presented. Many techniques have been developed by laypeople and are in common use but have not been evaluated scientifically.

### 3.1. Air Curtains, Fans, and Air-Handling Systems

Air curtains are produced by cylindrical fans mounted horizontally above doorways to produce a curtain of air with enough force (velocity) to prevent entry of insects. When air curtains are in operation, house flies can be observed flying less than 91 cm above the ground along the edges of the curtain of air, waiting for an opportunity to pass through. It has been demonstrated that air moving at 8 to 9.1 m/s [9,10] at 91 cm above the ground provides 80% exclusion of house flies. To achieve this desired velocity at knee level, air leaving the fan must be moving at a much higher velocity. Because many people dislike the strong airspeed at the head and shoulders level, the use of overhead air curtains operating properly is not popular. It was demonstrated that air curtain units mounted vertically on both sides of 91-cm wide doors and blowing across the doorway can prevent the entry of 98 to 100% of the house flies in a test facility with mean air velocities as low as 4 m/s [11].

Fans mounted above or blowing across fly congregation sites can prevent flies from entering the area (J.A.H., unpublished data). Because many fans have simple on and off switches, with a possibility of several speeds, trial and error is the best way to determine the necessary airspeed for the situation. Overhead fans are stationary, so the efficacious fan speed is dependent upon fan height. Rheostats simplify determining the proper speed. Finding the efficacious speed with portable fans also includes the ability to move the fan to the desired location.

Most commercial buildings are completely sealed from the outside environment, but have air handling systems that control the temperature and movement of air inside. These systems should be set to create a positive air flow so small volumes of air blow out of the buildings, especially when doors are opened. A negative air flow can pull insects inside when a door is opened (J.A.H., unpublished data). This is particularly important with large, self-opening doors that remain open for long periods during customer use.

### 3.2. Mesh Screen Windows and Mesh Screen and Slat Doors

Wire or plastic mesh screen has long been used to exclude house flies, but these materials are not commonly used in all parts of the world. Screen is a mechanical barrier so insecticide resistance in the flies is of no concern. Before World War II, 16 × 16 mesh screen was standard [12]. However, it was soon discovered that some species of mosquitoes, e.g., *Aedes* and *Anopheles* spp., could readily pass through [13]. Although 14-mesh screen would exclude house flies [14], it was suggested to use an 18-mesh screen to also preclude entry by smaller insects such as mosquitoes. In the early 1940s, screen with 18-mesh vertical wires and 14-mesh horizontal wires became the standard size still used today [12]. A discussion of air and light penetration of various mesh sizes is provided by Busvine [15].

Vinyl strip doors mounted across open doorways can be used to maintain temperature barriers and prevent entry of flies. Slats are clear and self-closing. Self-closing plastic mesh screen doors have been developed for use across standard width doorways of houses and aircraft [16].

## 4. Indoor Management

### 4.1. Ultraviolet Light Traps

**Figure 2:**
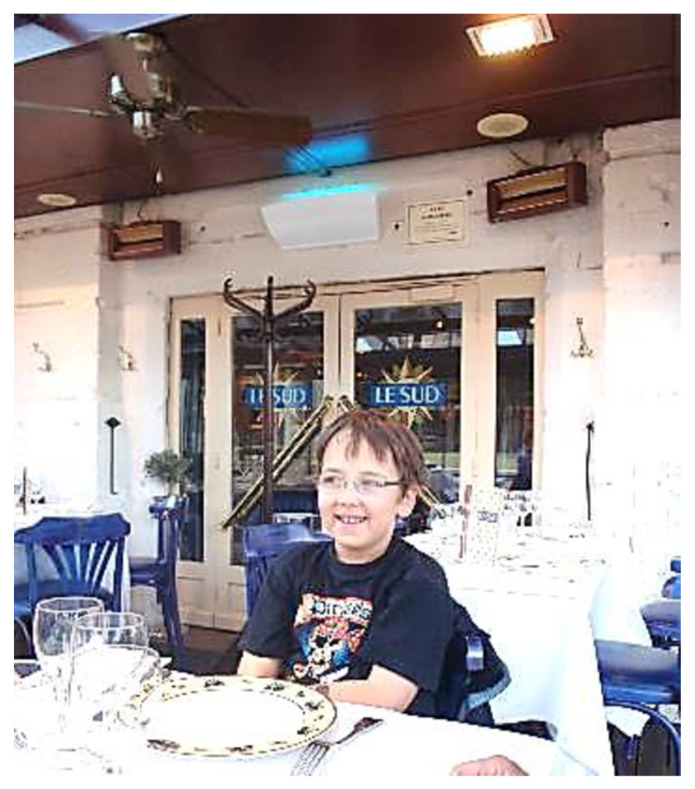
UV light trap mounted high above restaurant entry doors tends to attract flies to the doors with possible entry inside building, Lyon, France.

Traps with ultraviolet (UV) light are widely used for management of house flies in indoor urban and agricultural situations [17]. Some of the first traps had wire grids located in front of the UV fluorescent tubes to electrocute flies that were attracted to the lights [17]. It was noted [18,19] that flies killed by contacting the wires could fall beneath the trap; flies could also be blown away from the trap by the force of the electricity in the grid. Pickens [18] calculated the distances that electrocuted flies could be blown away from the trap. These findings clearly restricted the placement of these traps near food preparation areas where passive, non-toxic fly management is required. Additional research demonstrated that electrocution not only blew visible fly particles away from traps, but also caused the production of small airborne particles which could be potentially contaminated with pathogens such as bacteria and viruses [17,19,20]. This led to the development of UV light traps with glue boards to capture flies on sticky surfaces rather than fragmenting them through electrocution [20]. The significance of particle and pathogen dispersal from flies by the force of electrocution is not recognized internationally, and in many countries outside of the U.S. electrocutor grid traps can be seen mounted directly above food preparation areas (J.A.H., unpublished data) (Figure 2).

At present, all UV light traps with glue boards consist of a housing, UV fluorescent tubes, and glue boards. It has been shown that traps with open housings that allow the UV lights to shine directly into the room capture greater numbers of flies [21,22,23]. The UV fluorescent tube type can also influence trapping efficacy. Black light (standard UV tubes) and black light blue tubes both produce attractive peaks at 360–370 nm. However, the intensity of the black light tubes is greater than that of the black light blue tubes, which is probably why black light tubes attract more flies [21,22,23,24].

Glue board color has not been considered to be a factor influencing fly catch, and glue boards used commercially are usually white or black. Laboratory studies (J.A.H., unpublished data) indicated no difference between white and black glue boards. However, results from field studies showed that black glue boards reduced the number of flies captured by as much as 50% [25]. Some companies prefer black glue boards because the captured flies blend in and are not as visible to customers as they would be on white glue boards. More work is needed on the effects of glue board color. Some companies have tried to make their glue boards chemically attractive by adding pheromones or other attractive chemicals. These have not shown to be beneficial in laboratory studies [23]. Additionally, most climate-controlled commercial businesses have 5 to 8 complete air changes per hour which effectively eliminates most chemical odors in the building.

**Figure 3:**
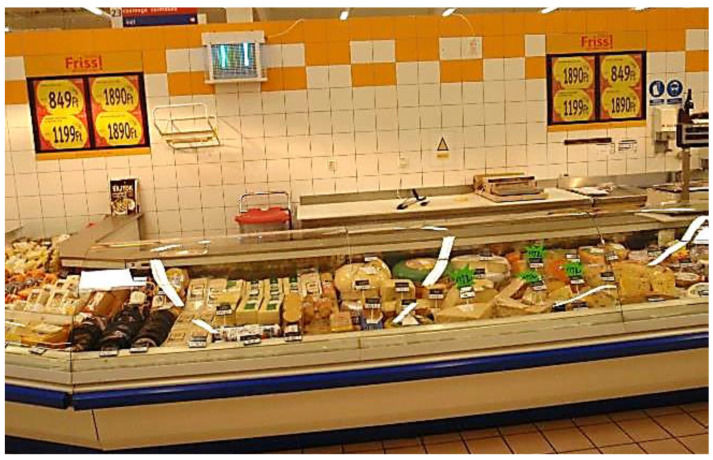
Supermarket meat and cheese counter with preparation area next to the wall and an electrocutor grid trap overhead, Budapest, Hungary.

**Figure 4:**
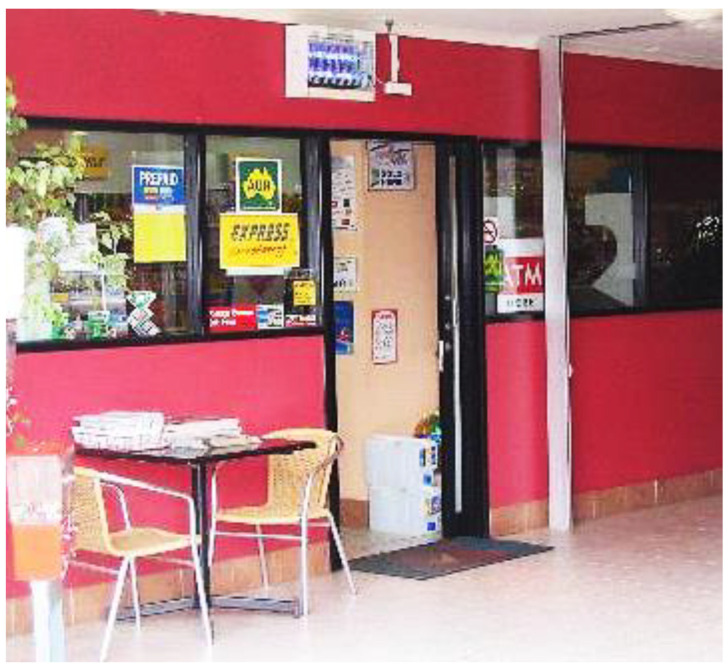
Zapper trap over a dining table. Restaurant door propped open.

Mounting height of ILTs is important but sometimes mounting at the optimum height is not an option, depending on activity near the desired site (Figure 3). Driggers [26] showed in open poultry houses that UV light traps mounted with the bottom edge of the traps ca.–10 cm above ground level captured 10 times more flies than those mounted with the bottom edge 1.5 m above ground level. House fly activity was greatest at and just after sunset, and the greatest activity was close to the ground. On several occasions food service employees stated similar observations. After stores are closed and overhead lights are dimmed, flies in the vicinity tended to congregate on the floor. If portable (MX-360) UV light traps were available, these were placed on the floor near the flies and many were captured (J.A.H., unpublished data). Evidence suggests that UV light traps placed on the floor capture more flies than those mounted 2 m above the floor [25], but more work is needed for clarification (Figure 4).

There is interest in making UV light traps more efficacious. In one field study it was shown that turning traps on and off at predetermined intervals enhanced trap catch [27]. This effect would not be popular in traps used commercially but perhaps this concept could be improved upon. Attempts have been made to change from fluorescent tubes to LEDs, but accounts of successful LED traps cannot yet be found in the literature.

There are many UV light traps on the market for indoor household use but the evaluation of only one can be found in the literature [28]. The performance of this trap was a great improvement over UV household traps made decades earlier (J.A.H., unpublished data). If other household UV light traps perform similarly, these types of traps might be beneficial for indoor fly management. More research is needed to evaluate additional trap models.

ILT manufacturers provide useful information on the most effective use of their products, so users should consult with manufacturers to ensure they are obtaining optimal performance from the device [29]. Certification organizations (such as AIB) stipulate fly control options, including recommendations for trap location, monitoring, maintenance, and use.

### 4.2. Window Traps and Window Stickers

Window traps and window stickers are devices placed in windows indoors to kill or capture flies that have been attracted to the windows from inside the building. The words trap and sticker are used almost interchangeably, and it is important to read the label to ascertain what is being purchased. Most of these devices, despite the name, capture insects on a non-toxic sticky surface. Sometimes the sticky surface is concealed in a decorative housing open at the top and applied directly to the windowpane to keep captured flies out of sight. The sticky surface itself can also be applied to the windowpane, without housing, leaving captured flies plainly visible.

Some devices consist of a surface that is toxicant-coated instead of sticky. Toxicants currently used, e.g., acetamiprid, must be consumed by the flies or other insects and may have sugar added to stimulate feeding (J.A.H., unpublished data). There is usually no housing, and the toxicant-coated surface is applied directly to the windowpane. Because these devices are not sticky, they remain free of flies. However, flies that die after feeding on the bait drop to and collect near the windowsill.

A trap with a completely different design is the Cluster Buster^®^ (Powder Trap Company, Ucluelet, British Columbia). It is essentially a reservoir containing finely powdered eggshells. The reservoir has an opening at the top that fits flush with the windowpane when the trap is placed low in the window on or above the sill. Flies above the trap that exhaust themselves after flying up and down the glass panes tend to fall into the trap through the slit, and sink in and are smothered by the powdered eggshells [30]. 

Despite various claims, the uniform attractant is the window. The comparative efficacy of these devices after the flies reach the window is unknown and needs to be determined.

### 4.3. Sticky Tubes and Ribbons

There are a variety of sticky tubes and ribbons for catching house flies. Few evaluations of these products have been published. Large sticky traps [31], tapes, and ribbons have been designed for agricultural use, but most are too large for the urban arena. Benefits of these devices are that they are inexpensive, easy to use, and pesticide-free. They are for indoor (under a roof) use only and designed to be placed high in a location where they can hang freely. Flies have a propensity to rest on vertically hanging objects [32], making these suspended devices attractive. The downside to these devices is that the captured flies are visible. This may or may not be important, depending on where the devices are deployed. There have been implications that flies captured on these devices attract additional flies. Thus, some brands of sticky tubes have black dots or simulated fly images printed on their surfaces. The effects of these images are controversial [33]. These devices have been used around, but not directly above, food processing areas. If some of these devices are placed too close to a heat source, the glue may liquefy and drip.

### 4.4. Timed-Release Aerosols (in Restrooms and Similar Locations)

Aerosol insecticides, usually pyrethrins, dispensed by battery-operated time-released units are available for use only in commercial urban fly control programs in food preparation and service areas, restrooms, and other rooms where flies might congregate. In some cases, all aerosol devices, toxicants and fragrances included, have been disabled or removed following complaints. One thing to consider when contemplating the use of time-released aerosol pesticides is the variation in house fly resistance nationwide to pyrethroids and other approved active ingredients. Scott et al. [34] found that survival ranged from 2.9 to 76% when house flies from 10 collections from nine different states were exposed to pyrethrins plus piperonyl butoxide. A benefit of pyrethrins is that there are limited residues that remain; however, insects must be within range of the aerosol dispenser to be affected. In many commercial buildings, where there are several complete air changes per hour [23], the life span of lingering aerosol droplets is probably brief.

### 4.5. Bags of Water with Coins

**Figure 5:**
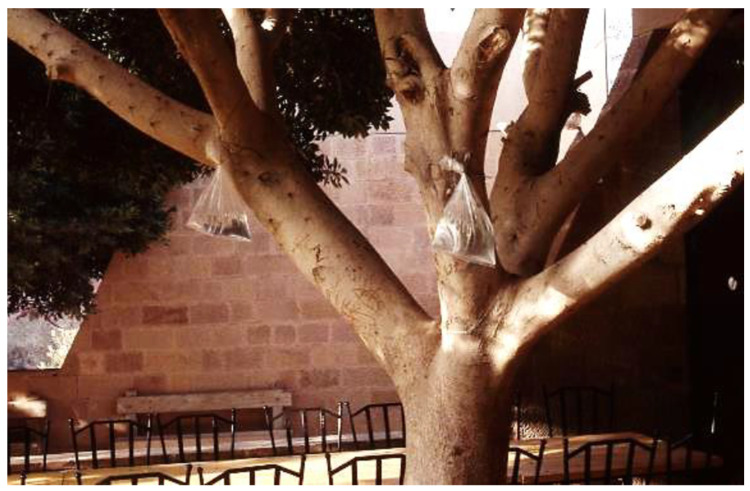
Bags of water for fly management hanging from a *Ficus* tree at an outdoor restaurant in Petra, Jordan, 1997.

The use of clear plastic bags containing water, with and without coins, to repel flies has been an interesting phenomenon that has continued to evolve. Originally, bags containing only water were used. In areas where Ziploc^®^ bags (S.C. Johnson and Son, Racine, WI, USA) were not available, similar bags were tied with twine and suspended under the eaves of a pole shed or other animal housing. Eventually the bags had to have two or three pennies inside, followed by a nickel. At some point between the addition of the pennies and the addition of the nickel, bag shape became important. Some adherents maintained the bags must have curved, not flat, bottoms. Others contended that the bottom of the bag must be flat. Some insisted that the bottom of the bag be completely horizontal to the ground while others said the bag should be hung from a top corner, so the bottom of the bag is at an angle to the ground. In Mexico and other countries in Latin America where windows have no screens, bags of water are hung from the top of an open window. In a restaurant, for example, a bag might be placed directly on the serving tables. Because one can assume that U.S. pennies and nickels might be difficult to obtain in some other countries, the local coinage must be a suitable substitute. It is impossible to know how far from the U.S. these bags have spread (assuming that the water bag development began in the U.S.), but the farthest documented sighting was in the city of Petra, Jordan, in 1997, where bags of water were hung in ficus trees (*Ficus benjamina*) around an outdoor restaurant (J.A.H., unpublished data) (Figure 5).

**Figure 6:**
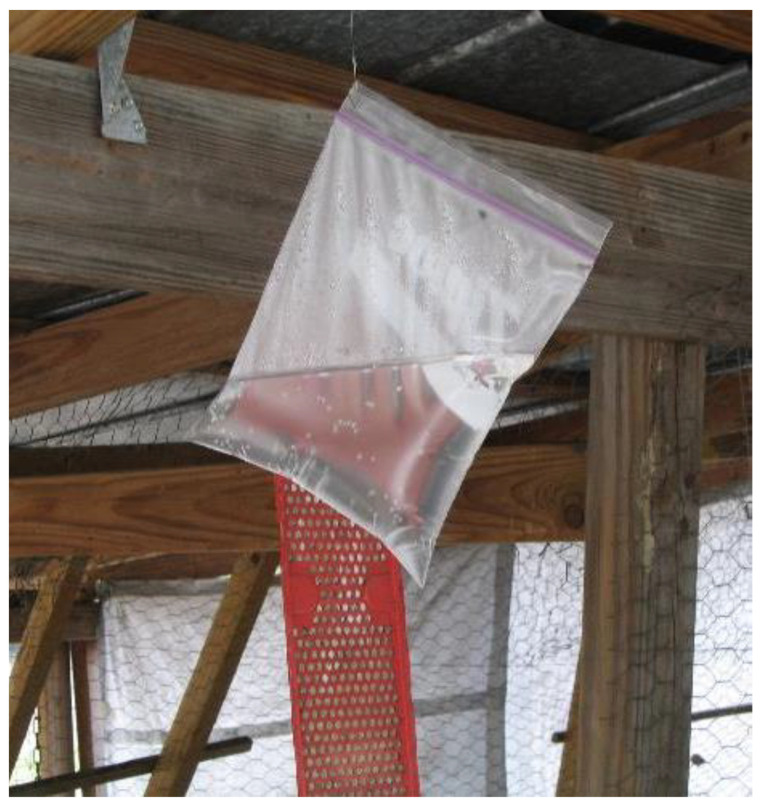
A bag of water without coins for fly management hanging in a pole shed near Newberry, Florida. A red QuikStrike Fly Abatement Strip can be seen hanging in the background.

There are many theories as to why bags of water repel flies, if they actually do. One theory is that flies see their reflection in the bags and are frightened away. Another theory concerns the manner in which light is reflected from the surface of the bags [35,36]. Documented field research on water bags has been published [37,38], but the authors were not really convinced that the bags repelled the flies. In a comparative study in the laboratory, Grützmacher and Nakano [39] found that clear bags containing yellow water attracted house flies but clear bags containing water with no added color repelled >30% of the house flies tested. More research is needed to substantiate the theories that attempt to explain the activities of the bags of water and to determine if house fly behavior when exposed to bags of water coincides with the theories (Figure 6). To date there are no definitive studies or data on the efficacy of water bags as a method of house fly control.

### 4.6. Fly Swatters

The fly swatter is such a ubiquitous mechanical fly killing device that it often goes unnoticed. When a fly is spotted indoors, some individuals will practically empty an aerosol can of an appropriate pesticide trying to kill it. A fly swatter and a little skill can also dispatch the fly. Fly swatters were developed in the 20th century [40] and have evolved from squares of metal window screen to plastic mesh designs. Although a fly swatter on the desk at the office seems a bit unappealing, keeping one handy but out of sight, in time of need, provides an easy pesticide-free way to kill an annoying fly quickly.

## 5. Sanitation to Eliminate Larval Habitats, Attraction of Adults, and Points of Fly Entry

House flies can be a major nuisance for residential and commercial accounts. Sanitation in urban areas can be described as the minimization of materials that are attractive to adult house fly populations. In the urban fringe (the transition zone where urban and rural uses mix) where various types of livestock/companion animals are permitted, managing materials that can be used for development of immature fly stages becomes an essential aspect of sanitation [41]. In metropolitan areas where housing of large animals is not permitted, larval habitats are more limited, and minimizing the attraction of adult populations becomes more important. Many times, odors produced by garbage receptacles signal food and oviposition sites to adult flies that arrive to find only odors. A similar effect can be created by grease spillage around dumpsters and grease disposal stations. Large trash compactors have exhaust fans to maintain a positive airflow from inside the food service facility. Trash compactor odors represent yet another odor plume. Restaurants and other food service businesses, e.g., supermarkets, exhaust cooking odors to the outside, producing large odor plumes that can be attractive not only to potential customers, but to flies as well (J.A.H., unpublished data). After flies arrive at these sites, the potential for fly entry increases.

Adult fly suppression in the urban fringe can be challenging if neighbors with livestock do not adequately manage fly breeding materials. Because house flies can generally disperse from 1 to 2 miles in less than 24 h [42,43] and their documented maximum range is 20 miles [44], management strategies in this situation involve trapping adults. 

Non-toxic fly traps lure flies into a container from which there is no escape. Typically, the flies then drown, if in a water trap, or die of starvation or overheating. Odors generated by decomposing fly carcasses attract more flies. Consumers appreciate that most of these traps are for single use and can then be disposed of, obviating the need for cleaning and handling the smelly mess.

Traps should be placed as a barrier and spaced about 30 feet apart and no more than 3 feet above the ground. Traps should not be placed near houses because they will attract flies to those areas. Commonly used outdoor traps involve a bag or jar containing a fly attractant which usually has a putrid smell, so these should be deployed at the periphery of the property to intercept flies without offending humans. When ready for use, water is added to the trap. These trap types remain active for about 7 days, are designed for large fly populations, and will not remain effective if they are catching only 10 to 15 flies per day. They work best if placed in sunny locations. In residential areas, where fly outbreaks may be an isolated event caused by organic wastes left in an uncovered trash receptacle, the removal and cleanup of breeding materials might suffice.

For commercial accounts, successful fly management outside may be difficult, and a sustainable active system, mainly for management of adults, must be implemented. Dumpsters should be kept closed and cleaned as often as possible. Dumpsters without tight closing lids can produce immature fly stages if conditions are suitable. Mature maggots may crawl out the top of the dumpster, drop to the ground and wander to pupation sites. Trash compactors are rarely cleaned in many locations because of cost, but a buildup of organic debris inside might allow for the development of flies.

Fly traps placed outdoors may be ignored by flies even though they are readily entering structures (J.A.H., unpublished data). In addition, in most cases trap placement is not optimal because of customer traffic patterns and esthetics. Most flies tend to enter through front doors [25] unless rear doors are left open for long periods. Front door entry areas should not be cluttered with vending machines, shrubbery, or any other objects that provide a resting place, shelter, or aggregation site for flies.

Fly exclusion fans [11] over rear doors used for unloading stock are often disabled by employees, which allows for fly entry. Fly exclusion fans over any door must be operating at the proper speed (9.1 m/sec 91 cm above the floor [10]) to be effective. Clutter can be used by flies as harborage and should be situated 10 to 12 feet away from rear entry doors to minimize adult fly entry. Residual pesticides and pesticide/growth regulator combinations are sometimes applied to outside walls near rear entry doors. Efficacy is difficult to determine.

Although PMPs may be limited in their ability to control house flies, they can focus on controlling situations that favor flies, addressing their efforts to environmental factors that support fly populations. No two situations are the same, but fly problems almost invariably include common elements of fly-attracting conditions (odors, moisture, lighting, etc.) and structural failures that permit fly entry.

## 6. Source Reduction

Source reduction is the elimination of materials in which fly eggs can be deposited and in which immature flies will develop to the next generation. An example of urban source reduction is the removal and proper disposal of decomposing organic food materials that might be used by fly maggots. On animal facilities source reduction involves substrates such as feed and feces. Proper waste removal minimizes fly production at that site and is an important feature of pesticide-free fly management. The adult flies in a particular area may be coming from a location where source reduction is not being practiced or where all breeding sites cannot be found and eliminated. If a fly production site can be identified, some negotiations might be needed to induce the owner to try some type of source reduction. Sometimes the use of small native parasitic wasps can be beneficial. These will be discussed below in the Biological Control section. Sometimes the source or sources from which adult flies originate cannot be located and the major management tools are fly traps.

## 7. Biological Control

Biological control involves use of beneficial organisms to manage pest species and can be another pesticide-free method to help suppress flies. Most biological control organisms produced and sold commercially for house fly management are for use against the immature stages. For best results, biological control is used where there is a constant source of fly development, or where such development is perceived. A horse stable in the urban fringe is a good example. There might be flies developing there most of the time, but it is impossible to be sure, so biological control organisms can be applied as an extra layer of protection. In most urban situations, residential and commercial, biological control is not feasible.

Biological control organisms include bacteria, e.g., *Bacillus thuringiensis* var. *israelensis* (Bti); nematodes, e.g., *Heterorhabditis* sp.; fungi, e.g., *Beauveria bassiana*; mites; insects such as the black dump fly, *Hydrotaea aenescens*; and several species of parasitic wasps [45]. By far the most successful and widely used of these are the parasitic wasps. Females of various species seek out developing fly pupae using chemical cues. Once found, the wasp stings the pupa to paralyze it and then deposits an egg on or in the pupa. After the egg hatches, the wasp larva slowly eats the immature fly, pupates, and emerges as an adult wasp [46].

Many of these tiny, gnat-size wasps (ca. 2–3 mm) are native and present in our environment, suppressing house fly populations. However, because of the house fly’s short developmental cycle (6.92 days at 33.2 °C) [47] and the wasp’s comparatively long developmental cycle (3 to 4 wks) [46], plus the parasitoid’s long processing time, it is difficult for these wasps in their native state to play more than a minor role in fly management. Commercial companies colonize parasitoid wasps and sell them in large numbers for inundative releases, so when they are released into the environment the larger wasp population will have a greater effect on the flies.

It was originally expected that wasps released in large numbers would self-propagate so subsequent releases of colonized wasps would not be required to maintain a high population level. Unfortunately, this does not happen, and additional releases must be made at regular intervals to maintain a high level of control throughout the fly season. Release of parasitoid wasps by themselves is not usually enough to effectively control flies. They must be used as part of an integrated pest management program that should also include sanitation.

Most biological control entities are effective only against immature stages, meaning that control must occur at the larval habitat before adult house flies emerge; seldom do PMPs have access to the habitat (and if they do, it should be removed and eliminated). The fungus *Entomophthora muscae* is effective against adult house flies, but it is slow-acting and limited by environmental conditions and insect behavior [48], so has a minimal role to play in urban house fly suppression. Biological control, of course, never eliminates the pest population; biological control organisms are adapted to use only a portion of the host population, allowing survival of hosts to ensure future parasitoid generations have suitable habitat. Therefore, biological control has little role to play in urban house fly suppression.

The Screwworm Eradication Program was very successful in eliminating screwworm (*Cochliomyia hominivorax* Coquerel) populations in the U.S. using the Sterile Insect Technique (SIT). This was accomplished by growing large numbers of screwworms in laboratory colonies and releasing sterilized males back into the environment. Like many flies, female screwworm flies mate only once. If they mate with a sterile male, they will not produce fertile eggs. Males mate numerous times and can therefore cause infertility in numerous females. Florida was declared screwworm-free in November 1959 [49] and the pest was eradicated from the U.S. in 1982 [50]. House fly populations are much larger than those of screwworm flies. To manage a pest population with SIT, sterile males must outnumber wild males by at least 10 to 1. In the latter part of the eradication program in Florida, up to 10,600 sterile screwworm flies were released per square mile [49]. If 10,600 sterile house flies were released in just one large poultry house they would be dwarfed by the exponentially larger wild population in the house (J.A.H., unpublished data). Therefore, extremely large numbers of sterile house fly males would need to be released to outnumber the wild males. Additionally, sterile males can be just as pestiferous as wild males. SIT will have to be modified to be effective and economically feasible for use against the high-density house fly.

## 8. Chemical Suppression

House flies are resistant to essentially every group of chemicals used against them [51], beginning with the organochlorine, DDT, and subsequently with most if not all insecticide classes up to the present. Resistance to imidacloprid was reported shortly after it was registered [52]. Resistance to insecticides with similar modes of action (cross resistance) is suspected. Very high resistance levels to pyrethroids, the most commonly used pesticide class for house fly management, have been found in many house fly populations [34]. Because of the propensity of house flies to rapidly develop resistance and the potential for efficacious products to become ineffective after a relatively short period, it has almost become an unspoken rule to limit broadcast application of insecticides for house fly control, concentrate on their focused use, and save the efficacious products for near emergency situations [1].

### 8.1. Toxic Baits

Toxic granular baits are available for localized treatment of adult house fly populations. Baits would rarely be necessary in an urban setting except at the urban fringe if horses or other livestock were nearby. Baits are sometimes used near rear entry doors of supermarkets, food service facilities, and restaurants if adult fly problems exist nearby. Baits must be applied in bait stations which can be placed in areas where adult flies congregate. Commercial bait stations are available, or bait stations can be fabricated from a plastic container with a flat bottom, e.g., a milk carton, cut with a 5- to 7.5-cm (2- to 3-inch) lip, or small disposable paper bowls. Whatever is used should be stabilized to prevent bait from spilling into and contaminating the surrounding environment. Bait stations should be placed out of the wind and weighted to prevent spillage. Some bait carriers pull water out of the air (deliquescence) and become semi-liquid by the end of the day. These are no longer active and should be disposed of as stated on the label; fresh bait should be applied on subsequent days if needed. Other bait carriers are not quickly affected by humid conditions and will remain active for several days.

The QuikStrike Fly Abatement Strip (Wellmark International, Schaumburg, IL, USA) is a self-contained bait station with several desirable qualities. The yellow toxicant (1% nithiazine) sugar-based matrix is applied to a strip of heavy white paper. The toxicant must be consumed by the flies to produce mortality. As the flies feed, the yellow strip turns white, indicating that the toxic bait has been consumed and the device should be replaced. Fly mortality usually occurs in 15 to 30 s (J.A.H., unpublished data). Thus, flies have time to consume only small quantities of the bait, which extends the longevity of the device. In a caged layer poultry house this device killed 30,000 house flies per hour in short trials (J.A.H., unpublished data) and remained active. This level of fly pressure would never be expected in the urban situation. According to the label, QuikStrike strips can be used “around commercial food-handling facilities, restaurants, bars, grocers, stores, and other facilities where house flies are a nuisance.” Additional uses are on the label. Despite what is stated on the label, the company recommends using it only outside in urban settings, and it should be mounted vertically on flat surfaces, e.g., walls, 4 ft or less above the floor, depending on access by children or animals. The bait is hygroscopic, so may melt at high humidity, and because the active ingredient is susceptible to degradation by ultraviolet radiation, the device should be placed out of direct sunlight exposure.

### 8.2. Larvicides (Including IGRs)

Chemicals classified as larvicides and Insect Growth Regulators (IGRs) are used strictly for control of the immature stages of the house fly [2]. Fly larvae often develop in the urban fringe where livestock or companion animals are found. Taylor et al. [53] applied the growth regulator, cyromazine (Neporex, 2-N-cyclopropyl-1,3,5-triazine-2,4,6-triamine), in granular form to decomposing hay residues, as might be found in the urban fringe. This successfully reduced numbers of stable fly, *Stomoxys calcitrans* L., larvae developing within. House fly larvae have been exposed since the 1980s to Larvadex, another form of cyromazine applied in poultry feed, and many house fly populations have developed Larvadex resistance [54,55]. Because Neporex (granular) is directly applied to maggot-infested habitat at a higher concentration than Larvadex, Neporex is effective against larval house flies [55]. Larvae might be found in the urban area, but this would be unexpected and should be handled on a case-by-case basis. For small, localized larval infestations, sanitation might be the easiest control method and would solve the problem long-term.

### 8.3. Adulticides

Adulticides can be applied as sprays, fogs, and baits. All forms of adulticide applications have been over-used for house fly management because of the ease of use and, in the past, effectiveness. Increasing costs and house fly resistance to insecticides have changed this outlook. Effective products should be used with care to prolong product life.

#### 8.3.1. Sprays

**Figure 7:**
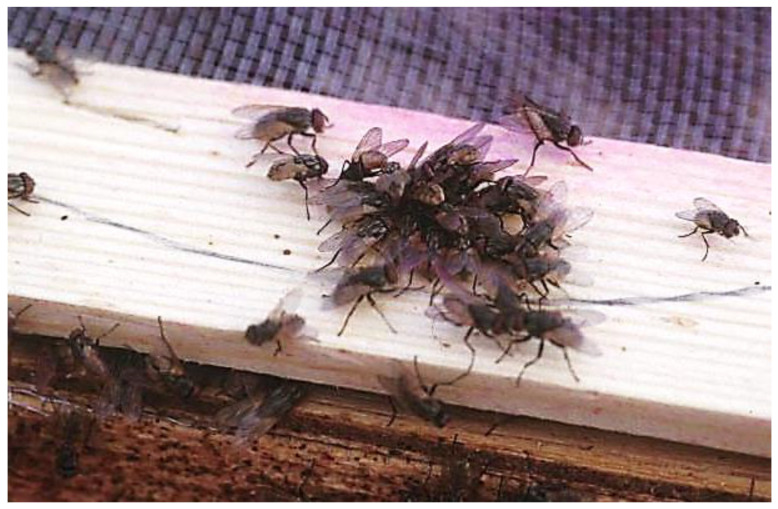
Target insecticides to congregation sites for adult house flies.

Products for spray application are formulated as a powder to be mixed with water, a liquid or emulsion to be diluted in water, or a ready-to-use liquid or aerosol. Except for most aerosols, liquid pesticides are intended to be applied as surface residuals to kill flies by contact. Residuals might remain effective from several days to several weeks, especially with microencapsulated formulations applied to nonporous surfaces, e.g., glass. Surface residuals might be needed rarely in the urban fringe, but they should be used only if fly populations are extremely large, and adults are congregating in known locations (Figure 7). Surface applications can quickly become covered in dust, e.g., in horse stalls, which renders them ineffective. Surface residuals may not be appropriate in urban areas unless there are problem sites that cannot be sufficiently cleaned. Some companies routinely spray the outsides of buildings and other structures and sometimes customers complain of overuse of pesticides. Surface residuals should be reserved for special cases and applied to surfaces where large numbers of adult flies are known to rest.

#### 8.3.2. Fogs 

Fogs are made to kill flies on contact and the proper droplet sizes are produced by foggers or spray nozzles designed for this purpose. Chemicals used for fogging are not usually designed to leave residuals. Automated overhead fogging systems have been used in animal facilities, e.g., horse stables, since the 1970s and the chemical of choice is still natural pyrethrins. Fog patterns can be altered by air movement, and fog may rarely reach the desired locations under windy conditions. In the urban area, hand-held foggers might be useful in situations where adult populations are extremely large and concentrated, e.g., in or around an improperly maintained dumpster, and a quick elimination of adults is required.

## 9. Conclusions

Urban pest management professionals recognize that house fly control is a multi-pronged undertaking that is based on sanitation, elimination (or at least reduction) of conducive conditions, and exclusion. Although insecticides often play a critical role, the tripartite base of any fly management scheme should be sanitation, source reduction, and exclusion.

As with any IPM program, house fly management includes monitoring to evaluate success of various components, allowing ongoing adjustment to optimize performance. Truly, with urban fly management there is no one-size-fits-all fly control program; every situation is different and requires strategies tailored to the particular setting and customer expectations.

**Figure 8:**
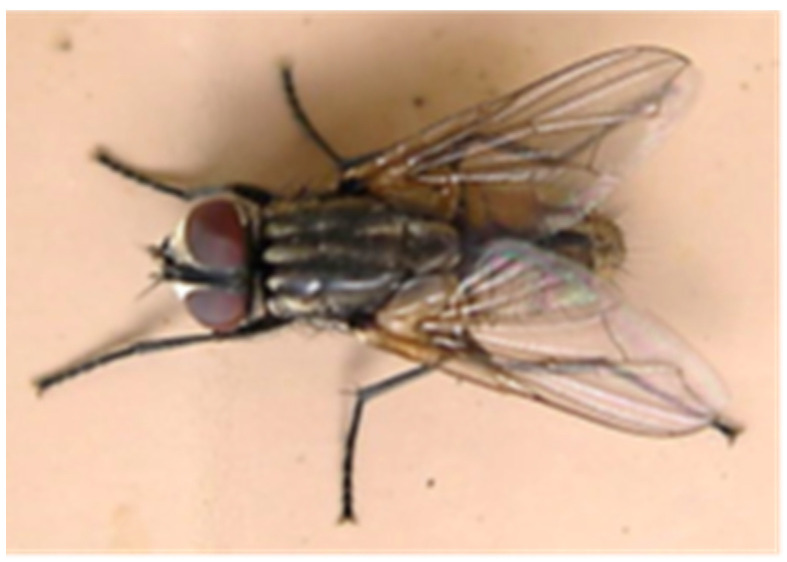
Adult house flies are targeted in urban pest management.

Because urban pest management has little opportunity to address immature fly stages, adults must be targeted and strategies customized for their elimination (Figure 8). Ideally, flies would be controlled as immatures, before they gain wings and disperse, but in urban settings PMPs generally must harvest adult flies that migrate from elsewhere, rather than implementing sustained population suppression [2].

With modern sanitation, house fly larval sites are limited in urban settings (decaying organic matter, feces, and household garbage), so long as systems function as intended. However, as has been shown by city ‘garbage strikes,’ a few days without scheduled garbage pickup can be disruptive, resulting in severe fly problems.

As was emphasized, fly exclusion from sensitive areas and from locations where people work, play, and live is a critical factor in pest management. Flies cannot be eliminated from the environment, but their access to our homes, businesses, and other structures can be limited.
